# Hygiene protocols for the treatment of denture-related stomatitis: local and systemic parameters analysis - a randomized, double-blind trial protocol

**DOI:** 10.1186/s13063-019-3854-x

**Published:** 2019-11-29

**Authors:** Adriana B. Ribeiro, Camila B. de Araújo, Luiz Eduardo V. Silva, Rubens Fazan-Junior, Helio C. Salgado, Aline B. Ribeiro, Caroline V. Fortes, Frank L. Bueno, Viviane C. de Oliveira, Helena de F. O. Paranhos, Evandro Watanabe, Cláudia H. da Silva-Lovato

**Affiliations:** 1 Department of Dental Materials and Prosthodontics, School of Dentistry of Ribeirão Preto, University of São Paulo (USP), Ribeirão Preto, Brazil; 2 Department of Physiology, Ribeirão Preto Medical School, University of São Paulo (USP), Ribeirão Preto, Brazil; 3 Department of Restorative Dentistry, School of Dentistry of Ribeirão Preto, University of São Paulo, Ribeirão Preto, Brazil

**Keywords:** Complete dentures, Biofilm, Cleansers, Candida, Stomatitis, Clinical trials, Heart diseases

## Abstract

**Background:**

Denture-related stomatitis (DS) is chronic multifactorial inflammation, strongly related to the presence of the biofilm that is the complex structure formed by microorganisms held together by a mucus-like matrix of carbohydrate that adheres to different surfaces, including the denture surface. DS has recently been correlated with deleterious cardiovascular alterations. The potential effect of hygiene protocols in the control of DS and randomized clinical trials that address this oral condition with cardiovascular complications are important in clinical decision-making.

**Material/design:**

A clinical trial, randomized, double-blind, and with parallel groups, will be conducted in Brazil The sample will consist of 100 patients without teeth in both arches, users of at least maxillary complete dentures, and diagnosed with DS, who will be allocated to groups (*n* = 25 per group) according to the different hygiene protocols: (1) brushing of the palate and immersion of the prosthesis in 0.25% sodium hypochlorite solution (positive control); (2) brushing of the palate and immersion of the prosthesis in 0.15% triclosan solution; (3) brushing of the palate and immersion of the prosthesis in lactose monohydrate; or (4) brushing the palate with citric acid and immersing the prosthesis in lactose monohydrate. The response variables will be heart rate variability and alteration of blood pressure (systemic level), remission of DS, removal of biofilm, reduction of microbial load (colony-forming units (CFU)), mouth and prosthesis odor level, expression of MUC1, proinflammatory cytokines, C-reactive protein (CRP), viscosity, pH and salivary flow (locally); patient-centred qualitative analysis will also be undertaken. Measurements will be performed at baseline and 10 days after the interventions. The results obtained will be statistically analyzed as pertinent, with a level of significance of 0.05.

**Discussion:**

This study will provide a guideline for clinical practice regarding the use of hygiene protocols in the treatment of oral diseases (DS) mediated by biofilm. Also, it may provide evidence of correlation of oral manifestation with cardiac risk.

**Trial registration:**

Brazilian Registry of Clinical Trials, RBR-4hhwjb. Registered on 9 November 2018.

## Background

The elderly population continues to expand, and today there are about 810 million people age 60 years and older in the world. By 2050, this number could reach 2 billion (22% of the global population). Intrinsic and extrinsic factors may promote decline in oral health, leading to tooth loss [1], and edentulism or complete tooth loss would be the final consequence of oral disease [2].

Complete dentures are a widely used option in the rehabilitation of the stomatognathic system [3] and may be associated with denture-related stomatitis (DS). *Candida albicans*, a common microorganism of the microflora of the oral cavity in humans, is often found in the biofilm of total dentures [4, 5]. However, in the presence of dentures and favorable conditions, such as biofilm, low salivary pH, regular sugar consumption and alterations in the local immune system (reduction of the activity of salivary antimicrobial enzymes, increase in transformer growth factor β and levels of nitric oxide), *C. albicans* becomes an opportunistic pathogen that leads to DS [6], and also may trigger halitosis [7].

DS is the most commonly found oral manifestation and the main indicator of poor oral health among the edentulous population, affecting one in three individuals using removable dentures [8]. DS is chronic multifactorial inflammation associated with the continuous use of maladaptive prostheses, hyposalivation, and poor hygiene; it is considered one of the main factors responsible for the evolution of inflammation due to the prevalence of *Candida* spp. [2, 9–12].

This inflammation can affect the individuals’ quality of life, since the clinical signs include erythema and edema of the palate mucosa, combined in some situations with subjective symptoms, such as dysgeusia (change in taste sensation) and burning sensation [8, 9, 13, 14]. However, in some patients, non-specificity of symptoms makes this disease often undiagnosed and untreated for long periods [15]. Furthermore, although considered to have little overall impact in terms of mortality/morbidity, early diagnosis and correct treatment may avoid potentiation of the immune response at other sites and/or systemic consequence [1, 16, 17].

Local inflammation associated, or not, with a biofilm, which is a complex structure formed by microorganisms held together by a mucus-like matrix of carbohydrate that adheres to different surfaces, including the denture surface [10], may trigger activation of monocytes and T cells, with overproduction of cytokines, such as interleukin (IL)-6, interferon γ, C-reactive protein (CRP) [17], tumor necrosis factor (TNF)-α and other proinflammatory cytokines, subsequently leading to atherosclerosis and hypertension, with increased cardiovascular risk [17, 18].

The relationship among stomatitis, *C. albicans* infection, and systemic inflammatory response is a recent finding and has not yet been clarified. Maciag et al. [16] analyzed peripheral blood immune cell activation to evaluate whether antifungal treatment of local inflammation caused by DS would influence the systemic immune response [16]. The authors did not find evidence of response complex immune mechanisms involved in the defense against oral fungal infection, though they verified a possible systemic inflammatory response to the topical application of nystatin, a macrolide polyene antifungal agent [17, 18]. Although transient and not intense, this effect should be considered a clinically important finding, since patients with DS are generally elderly, and as such, more susceptible to changes in the immune function. Since IL-1β is a proinflammatory cytokine in this susceptible population, even at a low level of production, this non-physiological outcome can influence the risk of inflammatory diseases, such as atherosclerosis or rheumatoid arthritis.

Osmenda et al. [17] evaluated the clinical relationship between DS treatment and endothelial dysfunction, as local inflammation in the oral cavity may cause the production of anti-inflammatory and proinflammatory cytokines, triggering systemic inflammation and activation of an immune response. The results indicate that DS treatment improved the endothelial function, the deterioration in which is known to precede the development of serious cardiovascular disorders, such as atherosclerosis and hypertension.

The evaluation of the predisposition of the individual with local inflammation to develop systemic diseases can be assessed through the detection of salivary mucins, which play an important role in the protection of buccal mucosa against mechanical and microbial aggression, and the residual saliva presenting higher amounts of mucins compared to total saliva. Recently, the correlation between salivary glycoprotein expression (MUC1, MUC5B, and MUC7) and buccal candidiasis has been suggested. These glycoproteins are responsible for the lubrication and protection of the oral tissues; they also can act in the modulation of the response of microorganisms [19, 20].

Glycosylated transmembrane mucins, such as MUC1, are known as the second line of defense, possibly acting as sensors for any disturbance in the environment, signaling this information into the cell [20, 21], and interacting with local bacteria [22], acting as a barrier for opportunistic infections, against several bacterial strains. At this time, the microorganisms of the dental biofilm are disseminated into the systemic circulation, through the invasion of the gingival tissue throughout the ulcerated epithelium [23]. Also, several proinflammatory cytokines, produced by local inflammation, can reach the systemic circulation [24]; thus, justifying the correlation between stomatitis and systemic diseases.

Heart rate variability (HRV) is one of the reliable and non-invasive approaches used to evaluate the autonomic control of the cardiovascular system in healthy individuals and in patients with the cardiovascular disorder [25]. Although there is no consensus on correlation between sympathovagal modulation and chronic inflammatory processes, there might be correlation between the vagus nerve and inflammation through two pathways. The first one is through the activation of the pituitary-hypothalamic-adrenal axis, resulting in systemic secretion of cortisol, which reduces the inflammation. The second one is through the vagal and sympathetic branches that reach the spleen, reflecting cholinergic and then noradrenergic signals, triggering splenic T cells via adrenergic receptors. These memory T cells will secrete the vagal neurotransmitter acetylcholine, which is responsible for the innate immune response, and which binds to the alpha-7 nicotinic acetylcholine receptor (nAChR) in monocytes, resulting in inhibition of the synthesis of inflammatory cytokines [26]. Together, these two pathways constitute the vagal anti-inflammatory reflex [27].

Scientific evidence of correlation among the biofilm, DS, cardiovascular disease, and adequate denture-related stomatitis treatment may contribute to the establishment of a protocol for the prevention and treatment of local inflammation, to be applied in primary care, which could significantly impact public health costs and patients’ quality of life. To our knowledge, this is the first proposal of a clinical, controlled, randomized, and double-blind study that proposes to test for correlation between treatment of DS, through hygiene and brushing solutions, and local and systemic inflammatory responses and cardiovascular impairment.

### Study hypothesis

The primary null hypothesis of the trial is that there is no difference between the protocols for the prevention and treatment of local and systemic inflammatory responses and cardiovascular risk.

## Methods

### Study setting

**Fig. 1:**
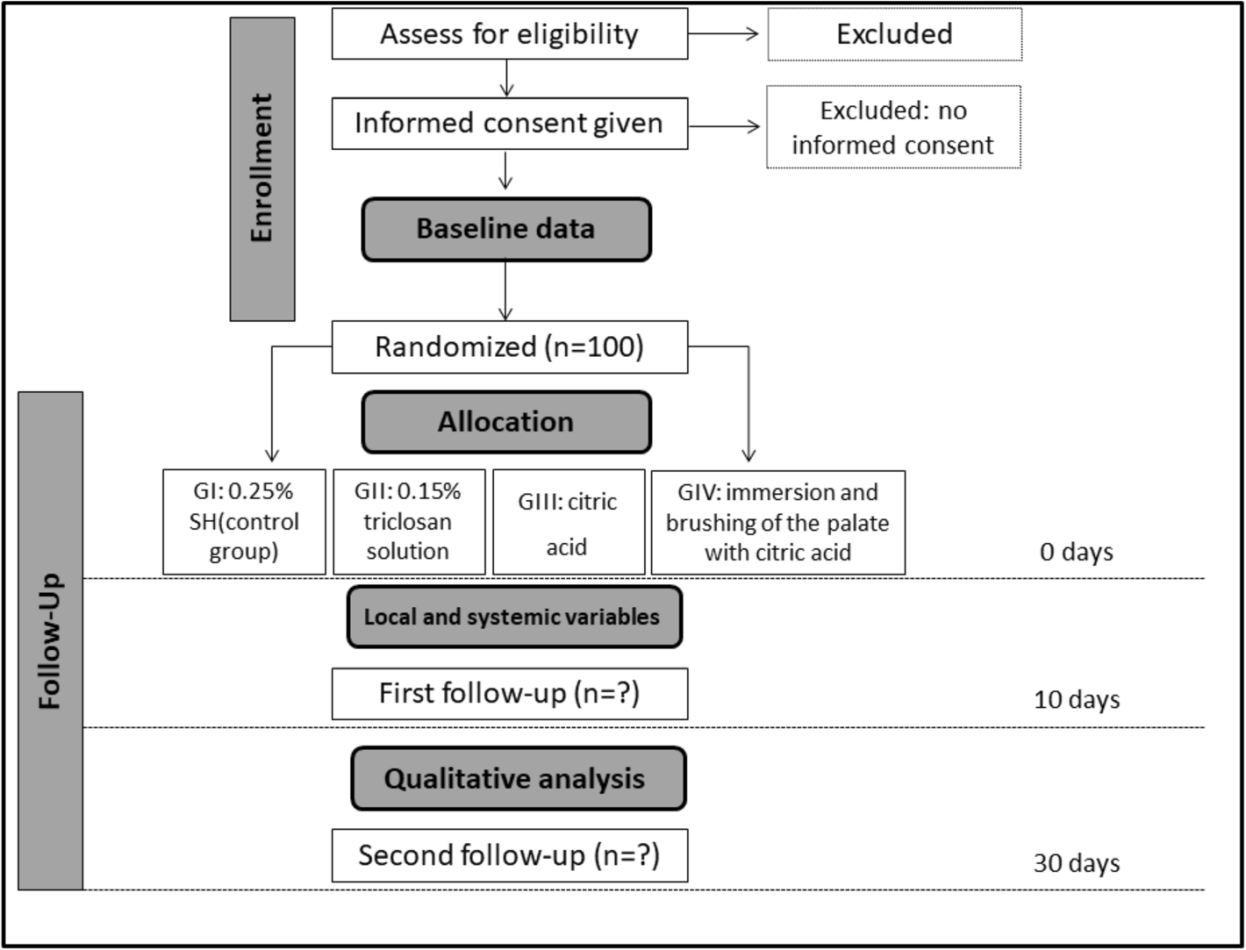
Flowchart of the randomized clinical trial (RCT) (adapted from the Consolidated Standards of Reporting Trials (CONSORT) statement). For each follow up, numbers of withdrawn and lost participants will be reported with reasons. G, group; SH, sodium hypochlorite

**Fig. 2:**
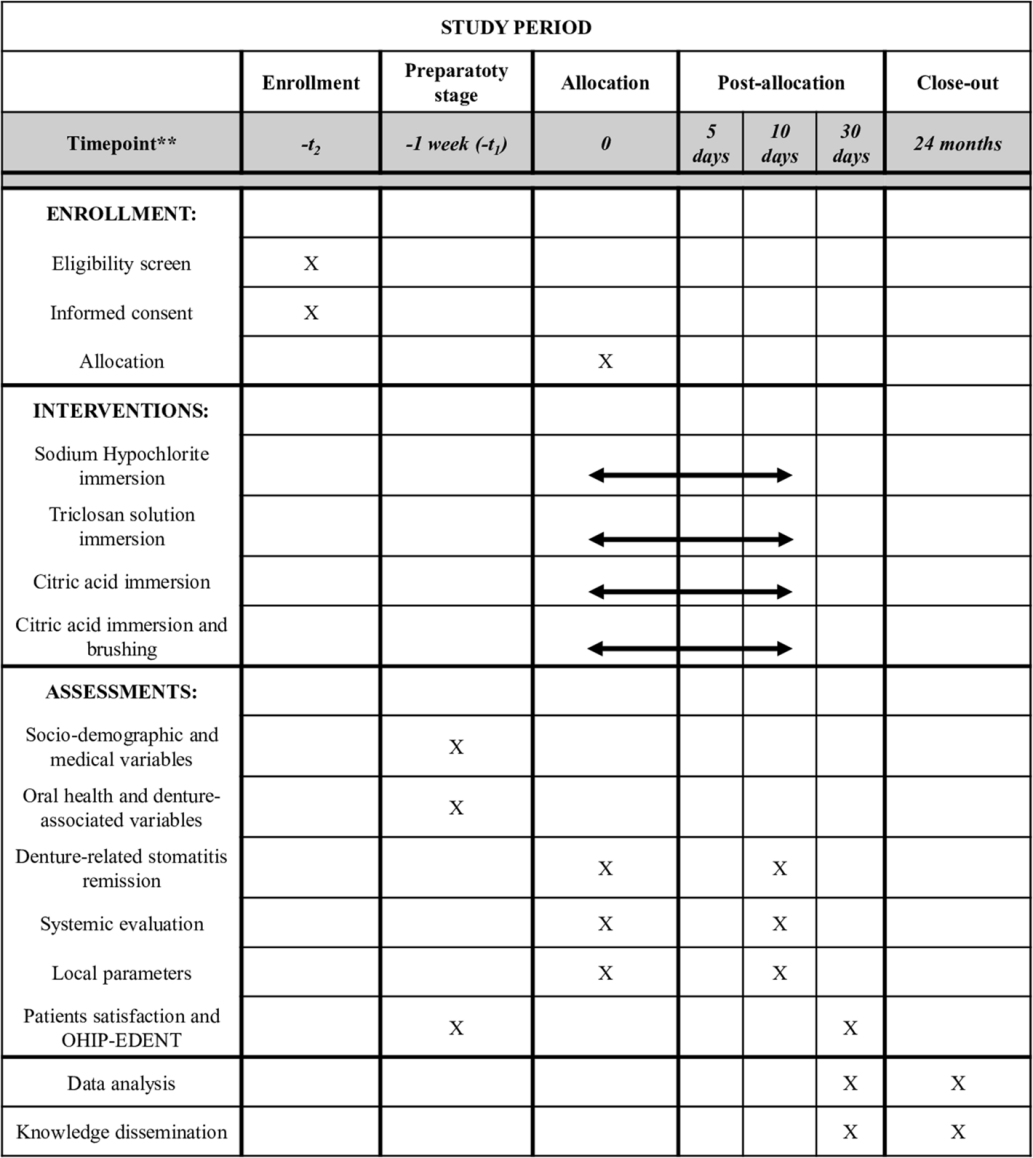
Study schedule: enrollment, allocation, baseline, interventions, and post-intervention assessments. OHIP-EDENT, Oral Health Impact Profile for edentulous people

A randomized, controlled, double-blind, clinical trial, with parallel groups named according to each hygiene protocol will be performed (Fig. 1). Figure 2 shows the study timeline, according to the Standard Protocol Items: Recommendations for Interventional Trials (SPIRIT) diagram. Additional file 1 presents the SPIRIT checklist.

The sample will be of convenience, consisting of patients with complete dentures who routinely attend the School of DEntistry of Ribeirão Preto - University of São Paulo (FORP/USP).

### Eligibility criteria

#### Inclusion criteria

The inclusion criteria are as follows: (1) patients may be of either sex; (2) patients must have good general health; (3) patients must be without teeth in both arches, users of upper and lower conventional complete prostheses, or, necessarily, users of a complete upper prosthesis (although edentulous mandibular) in good condition [28]; (4) prosthesis must be made from thermally polymerized acrylic resin and acrylic teeth; (5) patients must present with DS types IB, II, or III, according to the Newton Modified Classification [10]; and (6) their prostheses should present with biofilm with a score equal to, or greater than 1, according to Additive Index Exclusion criteria.

The following patients will be excluded: those patients who (1) present with prostheses with adaptation problems, failures, repairs, or fractures; (2) have allergy to any of the products studied; (3) have severe/serious illness that requires frequent hospitalization; (4) have systemic conditions favorable to the development of *Candida* spp.; (5) have used antibiotic, anti-inflammatory, or antifungal agents in the 4 weeks prior to the study; (6) have other lesions on the oral mucosa; (7) are already practicing palatal mucous brushing at recruitment; and (8) use replacement prostheses during the experimental period.

### Planned interventions

All patients will receive verbal and written instructions according to the hygiene protocols: they should brush the palate region with a soft toothbrush and water for 2 min, once a day (Toothbrush CS 5460C Adulto Ultra Macia, Curaprox, Curaden Swiss do Brasil Imp. Exp. LTDA, São Caetano do Sul, São Paulo, Brasil); immerse their dentures in the specific product once a day for the length of time proposed by the manufacturer; and brush the prosthesis for 2 min using a specific brush (Prosthesis Brush BDC150/152/153, Curaprox, Curaden Swiss do Brasil Imp. Exp. LTDA, São Caetano do Sul, São Paulo, Brazil) and neutral soap, three times a day. In addition, all patients will be instructed to remove the prosthesis during the night and leave it in a container with clean water, and in the morning to rinse the prosthesis under running water before inserting it into the oral cavity. Neutral soap and the solutions will be available to participants in identical dosing vials and in sufficient quantity for continuous use for 10 days; for greater control and monitoring of the hygiene protocol. citric acid in the form of an effervescent tablet will be removed from the package and placed in neutral packaging.

The parallel groups as determined by the different hygiene protocols will each consist of 25 participants. The protocols are as follows:

- Group 1 (G1 (control group)) - brushing the palate with a soft brush and prosthesis immersion in 0.25% sodium hypochlorite solution (control group)
- Group 2 (G2) - brushing the palate with a soft brush and prosthesis immersion in 0.15% triclosan solution
- Group 3 (G3) - brushing the palate with a soft brush and prosthesis immersion in citric acid (Nitradine, Bonyf AG, Liechtenstein, Switzerland)
- Group 4 (G4) - prosthesis immersion and brushing of the palate with citric acid and a soft brush (Nitradine, Bonyf AG, Liechtenstein, Switzerland)

The risk of adverse effects is low, although the use of palatal brushing may promote trauma in the case of excessive force. If the patient has a complaint about the products under investigation or adverse events, they may interrupt the treatment at any time, and notify us of what has happened. To verify if the patient is performing the protocol and minimizing any risks, there will be a consultation 5 days after the beginning of the treatment, for clarification of doubts and follow up. We expect good adherence due to the provision of an effective treatment modality as a major benefit combined with the need for routine clinical attendance at the School of Dentistry of Ribeirão Preto, and the fact that the provision will be made for post-trial care. We will also remind participants by telephone a few days before each follow-up appointment.

The patients will be instructed to avoid the consumption of beverages or foods that alter the metabolism, such as coffee, soda, alcohol, and chocolate, and to avoid physical exercise 24 h before the tests.

### Randomization, allocation, and blinding

The study will be double-blind, and to achieve this, each researcher (R) will have an assignment during the experiment: participants will be distributed into groups taking into account a random numerical sequence (on a 1:1:1:1 ratio) generated by a computer; a researcher (R1) who is not involved in the clinical steps will place the patients’ identification numbers in an envelope, using a blinded approach, and also will prepare the products. Another researcher (R2) will be responsible for opening the envelope at the moment of the delivery of the appropriate product to the patients, according to the hygiene protocols, and examine the patients and collect samples. A third researcher (R3) will distribute the protocols and orientate the participant, and finally the researcher R4 will perform blinded statistical analysis of the data. The researchers involved in the clinical steps (R2 and R3) and the patients will not be blinded because of the nature of the intervention. Patients’ allocated interventions will not be revealed until the statistical analysis is completed.

### Study outcomes

#### Primary: denture-related stomatitis remission

To evaluate the effect of the hygiene protocols on DS remission, the participants will be examined under the baseline condition and also 10 days after starting their specific use of the protocol. To quantify the inflammation, standardized photographs of the palate will be obtained (Digital Camera, Canon EOS, Canon EF 100 mm/2: 8 Macro Lens and Canon ML3 Circular Flash), with the focus centered on the median raphe region. The images will be transferred to the computer and two blinded, previously trained researchers will assign scores according to the classification of Kabawat et al. [10].

#### Secondary

##### Systemic evaluation

Patients’ blood pressure will be indirectly measured by the oscillatory sphygmomanometer method using an automated device (HEM7130, Omron Healthcare Brazil, São Paulo, SP); two to three measurements will be performed with a 5-min interval, recording the systolic (maximum) and diastolic (minimum) blood pressure. The technique for obtaining and classifying the individuals will follow the categorization proposed by the American Heart Association (2019).

Patients will be referred for continuous electrocardiographic monitoring with the Einthoven’s II lead, combined with monitoring of the respiratory rate with an elastic strap holding a stretch sensor around the thorax. The electrocardiogram and the respiratory sensor signal will be filtered (100 Hz to 0.5 kHz), amplified (BioAmp ADInstruments, Bella Vista Australia), digitalized (PowerLab 2/20 ADInstruments Bella Vista, Australia) and sampled (1000 Hz) continuously, using an IBM/PC. The files with the electrocardiogram recordings will be processed using a computer program (ECG Module for LabChart, ADInstruments, Bella Vista, Australia), which identifies the QRS complex of the electrocardiogram and calculates the duration of successive intervals between R waves (RR interval or cardiac interval). This processing will allow the generation of time series, beat-to-beat, from the cardiac interval values.

Cardiac interval variability will be analyzed using these recordings (spectral analysis). The series with RR interval values will be re-sampled at 3 Hz by cubic interpolation, to regularize the interval between beats. The series with interpolated RR interval values will be divided into segments with 512 values each, with a 50% overlap. The stationarity of each segment will be examined visually, and those with artifacts or transients will be excluded. Each segment will have its spectrum calculated by fast Fourier transform (FFT) after hanning windowing. The RR range spectra will be integrated into low frequency (LF) (0.04–0.15 Hz) and high frequency (HF) (0.15–0.50 Hz) bands. The relative power (normalized units) of the RR interval spectra in each frequency band, and the ratio of the LF and HF (LF/HF) powers thereof, will be determined.

Symbolic analysis searches for patterns of changes between successive cardiac interval values, classifies these changes and quantifies their occurrence. Sequences of three symbols will then be analyzed and classified into four different families, according to the number of variations found. The frequency of occurrence of each pattern will be analyzed and indicated as 0 V%, 1 V%, and 2 V%. The frequency of variations of type 0 V (sympathetic) and 2 V (vagal) are of interest as indicators of cardiocirculatory autonomic modulation.

The sampling entropy (SampEn) will be calculated from the IC series with the help of the JBioS software. The number of practical terms, SampEn quantifies the (logarithmic) probability that near-size patterns *m* will continue to *m* + 1. In other words, of the size patterns *m* that are similar, SampEn indicates which percentage of these will remain similar for *m* + 1, that is, when an extra point is considered. High probability of the patterns continuing closely indicates regularity, yielding low values of entropy. Cardiac variability and blood pressure will be recorded in the control period and after the treatment of DS through hygiene protocols. Thus, each patient will be their own control.

##### Local parameters

To verify biofilm removal on the inner surface of the upper prosthesis, the technique described will be performed according to Badaró et al. [11], such that from the biofilm evidence, the prostheses will be photographed in standardized positions. The areas of biofilm and the surface of the prosthesis will be calculated using software and will be applied in a formula to identify the amount of total area of the biofilm, before and after treatment.

The microbial load of the prostheses and the palate will be evaluated. Biofilm at these sites will be collected according to the protocol recommended by Kabawat et al. [10] and de Souza et al. [29]. Serial dilutions will then be obtained, which will be seeded in Petri dishes with culture medium specific for the growth of *Staphylococcus* spp. (Mannitol Salt Agar, Kasvi Imp. e Dist. de Prod. para Laboratórios Ltda, Curitiba, Brazil), Gram-negative bacteria (MacConkey Agar, Himedia Laboratórios PVI Ltd., Mumbai, India), *Candida* spp. (CHROMagar™ *Candida*, Becton Dickinson, Paris, France) and *Streptococcus mutans* (SB20 Modified Agar with Casitone, Himedia Laboratories PVI Ltd., Mumbai, India)*,* and incubated in a microbiological stove (De Leo Equipamentos Laboratoriais, Porto Alegre, RS, Brazil) at 37 °C for 48 h. *S*. *mutans* will be anaerobically cultivated in a microaerophilic environment in a jar (Permution, Curitiba, PR, Brazil). After the incubation period, the CFU count will be performed to quantify the microbial load. The biological specimens present in the collected biofilm will be stored at − 80 °C for future analysis, if utilized; we will formally communicate any amendments to the protocol during the trial.

The Breath Alert™ portable device (Tanita Corporation®-Japan), used according to the manufacturer’s instructions, will measure the odor of the cavity with and without the dentures. The odor level will be given as a score determined using the apparatus, with values that can vary from 1 to 4. Thus, the odor is classified according to the scores [30] as (1) odorless, normal; (2) mild, normal odor; (3) moderate, bad breath - perceptible; or (4) strong odor, noticeable. The patient will be evaluated for odor without the prosthesis in position and then with the prosthesis seated in the oral cavity. Thus, the odor related to the prosthesis will be calculated based on the difference between the odor of the cavity with and without the prosthesis.

Saliva samples will be collected to evaluate salivary parameters. The non-stimulated total saliva will be collected for 10 min by the method of spitting, which will be analyzed for viscosity and pH measured. The calibration of pH will be performed in a pHmetro (PHTEK, Curitiba, Paraná, Brazil) after calibration of the equipment. The kinematic viscosity of saliva will be measured using a glass viscometer, and the liquid viscosity coefficient will be calculated according to Shekhar et al. [31]. The total stimulated saliva will be collected for 5 min using the habitual chewing of 1 g of gum base [19], from which the calculation of saliva volume will be obtained to evaluate the salivary flow. Subsequently, saliva samples will be centrifuged at 10,000 × g for 15 min at 4 °C, to remove cellular debris. Aliquots of supernatant will be stored at − 80 °C for the analysis. The precipitates will be evaluated by ELISA [22] to identify and quantify MUC1 expression. The absorbance at 405 nm will be measured after 30–45 min in an ELISA reader.

As a control, wells without saliva will be used. The assay for saliva will be performed in triplicate, and the results will be presented as the mean difference between optical density (OD) readings in experimental and control wells. Salivary concentrations of cytokines (IL-6 and TNF-α) will be measured using ELISA kits (Multiplex Human Cytokine Magnetic Bead Kit (Millipore, USA) according to the manufacturer’s instructions [32]. The determination of C-reactive protein in saliva will be performed using CRP ELISA kits (Salimetrics Europe Ltda.) [33]. Both ELISA methodologies will be performed in duplicates on two standard 96-well microplates, according to the protocol provided by the respective suppliers.

##### Characterization of the sample

Socio-demographic characteristics of the study participants will be collected from medical and dental history reports during the first consultation. Information will be collected such as length of time of edentulism, age of the prostheses in use, drug profile, hygiene habits (use of oral antiseptics or chemical hygiene of prostheses, frequency of hygiene of the prostheses), continuous nocturnal use of prostheses, and smoking.

The quality of life associated with oral health will be evaluated by applying the Oral Health Impact Profile questionnaire, specific for edentulous patients (OHIP-EDENT), validated for the Brazilian population [34]. The questionnaire presents 19 questions in four domains: “complaints related to chewing”, “psychological discomfort and incapacity”, “social incapacity”, and “pain and mouth discomfort”. Participants will be asked to answer questions about how they feel using one of the following responses: “never”, “sometimes”, or “almost always”.

Patient satisfaction will be assessed by the frequency of specific symptoms, such as local pain, burning sensation, bad breath, and buccal dryness. The responses will be collected based on a 100-mm visual analog scale (VAS), which will provide parameters for assessing heterogeneity of the eligible sample at baseline. They will also be asked to give an open-label response to other sensory side effects [10, 29]. The quality of life associated with oral health and patient satisfaction will be collected at baseline and at 10 and 30 days after treatment with the hygiene protocols (Fig. 1).

During clinical examination, the condition of the prostheses in use, such as stability and retention according to Anastassiadou et al. [28], and biofilm deposits and visible debris, will be observed [11]. Data on the shape ridge and resilience of mucosabe collected. The data on quality of life related to oral health, quality of the prostheses, and general satisfaction will be collected for the characterization of the sample at baseline [29].

### Sample size estimation

Sample size for the quantitative outcomes was determined based on the primary outcome of this study (denture-related stomatitis remission). According to a previous trial [11], we used standard deviation of 2.19 (group 1 - saline group) and 1.79 (group 2 - sodium hypochlorite), a 95% confidence interval (bilateral), and a detectable difference of at least 2 logs. Based on power of 80%, this clinical study requires at least 21 participants. An additional 20% will be added to the planned sample to compensate for possible dropouts, thus resulting in a total of 25 participants.

### Statistical analysis

Data entry and analysis will be conducted in a blinded fashion. The data collected for the groups, prior to adherence to the hygiene protocols, such as age, gender, and grade of schooling, will be compared to ascertain initial similarity. The effect of groups on primary and secondary outcomes will be assessed. When applicable (i.e OHIP-EDENT), pre-treatment values will be applied as a co-variable in the statistical model. The significance level of the tests will be 0.05. Noncompliant participants will be followed up, such as those requiring interruption of one of the hygiene protocols, and to evaluate the significance of protocol deviations. In other words, the statistical analysis will consider the participants according to the treatment received (per protocol), as well as according to the planned treatment (intention to treat (ITT)); the results will be compared.

The data will be analyzed for homogeneity; in the case of non-normal distribution, the Brunner Langer nonparametric test will be used. For the categorical variables (questionnaire) the Friedman test will be applied to compare the different time points, and the Kruskal-Wallis test will be applied to compare the groups. Correlation between the quality of life indices and the quantitative variables will be tested (Pearson correlation coefficient). The Tukey test with Bonferroni adjustment will be used for subgroup analysis. The Dunn post-test test will be used with the Kruskal-Wallis test.

A flowchart of the participants will be prepared for a detailed explanation of the characteristics of the sample and the quantification of quitters and missing participants. This part will provide the number of individuals examined, reasons for exclusion, and the number of recruited, treatment-allocated participants who complete the trial and are included in analysis at the study end. The flowchart will provide the reasons for any deviation from the protocol.

### Data management, monitoring, and auditing

A data monitoring committee of independent researchers will check collected data regularly. This researcher shall have no relationship with the trial sponsors. Moreover, the Institutional Board at Sao Paulo University may conduct an independent audit at any time. Our study will end when the target sample size is reached or on the scheduled date of closure.

### Ethical considerations and dissemination

This study protocol was approved by the Research Ethics Committee of the School of Dentistry of Ribeirão Preto (CAAE 93712418.1.0000.5419), and registered on 9 November 2018, on the ReBec platform (http://www.ensaiosclinicos.gov.br/rg/?q=RBR-4hhwjb) and will be reported in compliance with the Consolidated Standards of Reporting Trials (CONSORT) statement.

Eligible candidates will be invited to participate in the study and given sufficient time to read the informed consent and ask any questions pertaining to their participation. After signing the consent form (Additional file 2), the participants will be formally enrolled in the study and baseline assessment will be conducted. As a consent clause, we will grant the individuals the right to withdraw from the study at any time. All documents relating to the participants, such as terms of consent and clinical data, will be kept in a locked cabinet to guarantee their confidentiality.

On the consent form, participants will be asked if they agree to use of their data should they choose to withdraw from the trial. Participants will also be asked for permission for the research team to share relevant data with people from the School of Dentistry of Ribeirão Preto, University of São Paulo (USP), Ribeirão Preto, Brazil taking part in the research or from regulatory authorities, where relevant. This trial does involve collecting biological specimens for storage.

Electronic data handled by the researchers will contain numerical codes in place of the names. Any changes to the protocol will be made after seeking the opinion of the Research Ethics Committee and development agencies. Also, the authors will disclose the results of this proposal, regardless of the findings. The results of the randomized clinical trial (RCT) will be presented at major scientific conferences, including the International Association for Dental Research (IADR) General Session; and will be disseminated in a peer-reviewed journal.

## Discussion

This RCT will provide a guideline for clinical practice on the use of hygiene protocols in the treatment of oral diseases mediated by biofilm (DS). Also, it may provide evidence of correlation between oral manifestation and cardiac risk.

DS has been recently associated with systemic implications (variations in blood pressure and endothelial dysfunction) that precede the development of serious cardiovascular disorders, such as atherosclerosis and hypertension, which are changes in general health with high mortality/morbidity rates [16–18]. However, the mechanisms that are related to correlation between oral inflammation and cardiovascular effects are not yet fully described; one of the most important hypotheses is the pre-activation of the immune system [17].

The information from this study will also improve clinical decision-making and potentially protect edentate patients from harm caused by ineffective treatment, and the incorporation of hygiene protocols for oral tissues and prostheses can achieve favorable results associated with low costs and minimal adverse effects, besides possibly avoiding the involvement of opportunistic diseases, which may lead to a decreased risk of cardiac diseases. The resulting published information will provide evidence for the development of clinical recommendations for DS, which will not only be used for publication in indexed journals but also for public health information services.

Thus, we predict a favorable impact on public health, while the results will provide the basis for future investigations of the characterization of possible changes in HRV, correlated or not, with changes in blood pressure, salivary proinflammatory markers and salivary parameters with DS. Given the vast prevalence of DS in this elderly population, such knowledge may be crucial for effective control as well as for the detection of cardiovascular disease risk.

## Trial status

First version (01) of the study protocol, 22 July 2019.

Second version (02) of the study protocol, 17 October 2019.

Recruitment of patients: Initial date 03 September 2018

This trials is currently analysing clinical results and will initiate the cytokines and MUC1 analyses.

## Supplementary information

**Additional file 1.** The Standard Protocol Items: Recommendation for Interventional Trials (SPIRIT) checklist for this trial.

**Additional file 2.** Model consent form.

## Abbreviations

CFU: Colony-forming unit
CONSORT: Consolidated Standards of Reporting Trials
CRP: C-reactive protein
DS: Denture stomatitis
ELISA: Enzyme-linked immunosorbent assay
HRV: Heart rate variability
ITT: Intention to treat
MUC: Transmembrane mucin
OD: Optical density
OHIP-EDENT: Oral Health Impact Profile for edentulous people
RCT: Randomized clinical trial
SampEn: Sampling entropy
SPIRIT: Standard Protocol Items: Recommendations for Interventional Trials
VAS: Visual analog scale

## References

[1] Hannah VE, O’Donnell L, Robertson D, Ramage G (2017). Denture stomatitis: causes, cures and prevention. Prim Dent J.

[2] Kulak-Ozkan Y, Kazazoglu E, Arikan A (2002). Oral hygiene habits, denture cleanliness, presence of yeasts and stomatitis in elderly people. J Oral Rehabil.

[3] Regis R. R., Cunha T. R., Della Vecchia M. P., Ribeiro A. B., Silva-Lovato C. H., de Souza R. F. (2013). A randomised trial of a simplified method for complete denture fabrication: patient perception and quality. Journal of Oral Rehabilitation.

[4] Campos M. S., Marchini L., Bernardes L. A. S., Paulino L. C., Nobrega F. G. (2008). Biofilm microbial communities of denture stomatitis. Oral Microbiology and Immunology.

[5] Ghannoum Mahmoud A., Jurevic Richard J., Mukherjee Pranab K., Cui Fan, Sikaroodi Masoumeh, Naqvi Ammar, Gillevet Patrick M. (2010). Characterization of the Oral Fungal Microbiome (Mycobiome) in Healthy Individuals. PLoS Pathogens.

[6] Gasparoto Thaís H., Sipert Carla R., de Oliveira Carine E., Porto Vinicius C., Santos Carlos F., Campanelli Ana P., Lara Vanessa S. (2011). Salivary immunity in elderly individuals presented with Candida-related denture stomatitis. Gerodontology.

[7] Garrett Neal R. (2010). Poor Oral Hygiene, Wearing Dentures at Night, Perceptions of Mouth Dryness and Burning, and Lower Educational Level May be Related to Oral Malodor in Denture Wearers. Journal of Evidence Based Dental Practice.

[8] Gendreau Linda, Loewy Zvi G. (2011). Epidemiology and Etiology of Denture Stomatitis. Journal of Prosthodontics.

[9] Kossioni Anastassia E. (2010). The prevalence of denture stomatitis and its predisposing conditions in an older Greek population. Gerodontology.

[10] Kabawat Marla, Souza Raphael, Badaró Maurício, Koninck Louis, Barbeau Jean, Rompré Pierre, Emami Elham (2014). Phase 1 Clinical Trial on the Effect of Palatal Brushing on Denture Stomatitis. The International Journal of Prosthodontics.

[11] BADARÓ Maurício Malheiros, SALLES Marcela Moreira, LEITE Vanessa Maria Fagundes, ARRUDA Carolina Noronha Ferraz de, OLIVEIRA Viviane de Cássia, NASCIMENTO Cássio do, SOUZA Raphael Freitas de, PARANHOS Helena de Freitas de Oliveira, SILVA-LOVATO Cláudia Helena (2017). Clinical trial for evaluation of Ricinus communis and sodium hypochlorite as denture cleanser. Journal of Applied Oral Science.

[12] Felipe Lorena de Oliveira, Júnior Willer Ferreira da Silva, Araújo Katialaine Corrêa de, Fabrino Daniela Leite (2018). Lactoferrin, chitosan and Melaleuca alternifolia —natural products that show promise in candidiasis treatment. Brazilian Journal of Microbiology.

[13] Abaci Ozlem, Haliki-Uztan Alev, Ozturk Berran, Toksavul Suna, Ulusoy Mubin, Boyacioglu Hayal (2010). Determining Candida spp. Incidence in Denture Wearers. Mycopathologia.

[14] Salerno C, Pascale M, Contaldo M, Esposito V, Busciolano M, Milillo L (2011). Candida-associated denture stomatitis. Med Oral Patol Oral Cir Bucal.

[15] Le Bars Pierre, Kouadio AlainAyepa, N'goran JustinKoffi, Badran Zahi, Soueidan Assem (2015). Relationship between removable prosthesis and some systemics disorders. The Journal of Indian Prosthodontic Society.

[16] Maciąg J, Mikołajczyk T, Matusik P, Nowakowski D, Robertson D, Maciąg A, Osmenda G, Cześnikiewicz-Guzik M (2017). The effect of treatment of denture-related stomatitis on peripheral T cells and monocytes. Oral Health Prev Dent.

[17] Osmenda Grzegorz, Maciąg Joanna, Wilk Grzegorz, Maciąg Anna, Nowakowski Daniel, Loster Jolanta, Dembowska Elżbieta, Robertson Douglas, Guzik Tomasz, Cześnikiewicz-Guzik Marta (2017). Treatment of denture-related stomatitis improves endothelial function assessed by flow-mediated vascular dilation. Archives of Medical Science.

[18] Maciąg Joanna, Osmenda Grzegorz, Nowakowski Daniel, Wilk Grzegorz, Maciąg Anna, Mikołajczyk Tomasz, Nosalski Ryszard, Sagan Agnieszka, Filip Magdalena, Dróżdż Mirosław, Loster Jolanta, Guzik Tomasz J., Cześnikiewicz-Guzik Marta (2014). Denture-Related Stomatitis Is Associated with Endothelial Dysfunction. BioMed Research International.

[19] Chang Woon-Ic, Chang Ji-Youn, Kim Yoon-Young, Lee Gene, Kho Hong-Seop (2011). MUC1 expression in the oral mucosal epithelial cells of the elderly. Archives of Oral Biology.

[20] Kho H-S (2018). Oral epithelial MUC1 and oral health. Oral Diseases.

[21] Kang Jeong-Hyun, Kim Yoon-Young, Chang Ji-Youn, Kho Hong-Seop (2017). Relationships between oral MUC1 expression and salivary hormones in burning mouth syndrome. Archives of Oral Biology.

[22] Gabryel-Porowska H, Gornowicz A, Bielawska A, Wójcicka A, Maciorkowska E, Grabowska SZ, Bielawski K (2014). Mucin levels in saliva of adolescents with dental caries. Med Sci Monit.

[23] Arimatsu K, Yamada H, Miyazawa H (2014). Oral pathobiont induces systemic inflammation and metabolic changes associated with alteration of gut microbiota. Sci Rep.

[24] Preshaw Philip M., Taylor John J. (2011). How has research into cytokine interactions and their role in driving immune responses impacted our understanding of periodontitis?. Journal of Clinical Periodontology.

[25] Aghdasi-Bornaun H, Kutluk G, Keskindemirci G, Öztarhan K, Dedeoğlu R, Yılmaz N, Tosun Ö (2018). Evaluation of autonomic nervous system functions in frame of heart rate variability in children with inflammatory bowel disease in remission. Turk J Pediatr.

[26] Rosas-Ballina M., Olofsson P. S., Ochani M., Valdes-Ferrer S. I., Levine Y. A., Reardon C., Tusche M. W., Pavlov V. A., Andersson U., Chavan S., Mak T. W., Tracey K. J. (2011). Acetylcholine-Synthesizing T Cells Relay Neural Signals in a Vagus Nerve Circuit. Science.

[27] Gidron Yori, Deschepper Reginald, De Couck Marijke, Thayer Julian, Velkeniers Brigitte (2018). The Vagus Nerve Can Predict and Possibly Modulate Non-Communicable Chronic Diseases: Introducing a Neuroimmunological Paradigm to Public Health. Journal of Clinical Medicine.

[28] Anastassiadou V, Naka O, Heath MR, Kapari D (2002). Validation of índices for funtional assessment of dentures. Gerodontology..

[29] de Souza RF, Khiyani MF, Chaves CAL, Feine J, Barbeau J, Fuentes R, Borie E, Crizostomo LC, Silva-Lovato CH, Rompre P, Emami E. Improving practice guidelines for the treatment of denture-related erythematous stomatitis: a study protocol for a randomized controlled trial. Trials. 2017. 10.1186/s13063-017-1947-y. 10.1186/s13063-017-1947-y PMC5420092 28476133

[30] Pedrazzi V, Sato S, de Mattos Mda G, Lara EH, Panzeri H (2004). Tongue-cleaning methods: a comparative clinical trial employing a toothbrush and a tongue scraper. J Periodontol.

[31] Shekhar A, Das S, Bhattacharyya J, Goel P, Majumdar S, Ghosh S (2018). A comparative analysis of salivary factors and maxillary denture retention in different arch forms: an in vivo study. J Indian Prosthodont Soc.

[32] Ramírez-Amador V, Zambrano JG, Anaya-Saavedra G, Zentella-Dehesa A, Irigoyen-Camacho E, Meráz-Cruz N, Ponce de León-Rosales S (2017). TNF as marker of oral candidiasis, HSV infection, and mucositis onset during chemotherapy in leukemia patients. Oral Dis.

[33] Justino Celine IL, Duarte Kátia, Lucas Susana, Chaves Paulo, Bettencourt Paulo, Freitas Ana Cristina, Pereira Ruth, Cardoso Susana, Duarte Armando C, Rocha-Santos Teresa AP (2014). Assessment of cardiovascular disease risk using immunosensors for determination of C-reactive protein levels in serum and saliva: a pilot study. Bioanalysis.

[34] Souza RF, Patrocínio L, Pero AC, Marra J, Compagnoni MA (2007). Reliability and validation of a Brazilian version of the Oral Health Impact Profile for assessing edentulous subjects. J Oral Rehabil.

