# Supplementary material for: Hygiene protocols for the treatment of denture-related stomatitis: local and systemic parameters analysis - a randomized, double-blind trial protocol
Source: Trials. 2019 Nov 29;20:661. doi: 10.1186/s13063-019-3854-x (PMC6884795; doi:10.1186/s13063-019-3854-x)
Supplement: Supplementary file 2 — Additional file 2. Model consent form. [file 13063_2019_3854_MOESM2_ESM.pdf]

UNIVERSITY OF SAO PAULO
DENTAL SCHOOL OF RIBEIRÃO PRETO

FREE AND EXCLUDED CONSENT TERM

(Chapter IV, items 1 to 3 of Resolution 466/2012 - National Health Council)

We invite you ____, General identity record ____________________, to participate as a volunteer in the research "Clinical, microbiological and patient-centered assessment of different protocols for hygiene to complete denture. Randomized clinical study "developed under the responsibility of Prof. Dr. Cláudia Helena Lovato da Silva. After receiving all the necessary explanations for the participation of the research, it is up to you to decide, of its own free will, if you want to participate and sign this Term of Free and Informed Consent with the purpose of authorizing your participation in said research. The researchers responsible for presenting this document and obtaining their consent will be Adriana Barbosa Ribeiro, Camila Borba de Araújo and Frank Lucarini Bueno, and Prof. Dr. Cláudia Helena Lovato da Silva will be responsible for evaluating the treatment performed.

Denture cleaning should be performed in order to decrease the risk of mouth infections and improve the quality of life of denture users. This study aims to analyze different denture cleaners and to observe its effects on the cure of prosthesis-related stomatitis, which is an inflammation commonly found in the oral cavity related to the presence of microorganisms and debris accumulation in the prostheses, which can lead to local and systemic implications.

You are being invited, among the patients that are already undergoing treatment in the clinic of Total Prosthesis of the Faculty of Dentistry of Ribeirão Preto-USP (FORP) and that fit the objective of this study, to participate in the research. Regardless of whether you agree to participate or not, you will continue the treatment offered to you at the FORP clinic without any alteration or impairment in your care.

To participate in this survey, you will need to follow the denture and oral cavity guidelines for 10 days. To clean the dentures you should immerse them in 200 ml of the products offered for 20 minutes, once a day. To clean the palate you should brush the area with the special brushes provided. All materials will be provided to you during this time and throughout the research you should answer a questionnaire about your oral health related quality of life. Hygiene materials will be provided during the research.

During the return visits, at the clinic, we will collect material from the upper dentures and the palate with swabs, as well as taking pictures of the oral cavity and the denture, and the saliva will be collected by the spit method, as well as electrocardiogram examinations and blood pressure measurement. A measurement of the level of halitosis (smell) will also be carried out by means of a syringe which, after capturing the air from your mouth and the denture, will be lead in an apparatus. These tests will not cause you any complications or damage, as they will not cause pain or injury.

This study has nothing to do with your treatment at FORP. You participant who agrees to participate in this research project will have no additional cost. All costs related to this study (materials for plaque collection, clinical analysis and microbiological analyzes) will be borne by researchers.

The risks and discomforts of participating in this study are minimal, for example, the possibility of injuring the palate if you perform a very strong brushing. However, if you feel any discomfort when using the products offered, you can stop using them at any time and the possible effect will be evaluated by the researchers. As a benefit, prosthesis disinfection and cavity hygiene will provide you with more health and relief of stomatitis-related symptoms. Also this study will be the identification of the best method of hygiene / decontamination of prostheses, reducing in this way the risk of development of infections and impossibility of using the prosthesis.

The procedures will be performed during routine care appointments. If there is a need for exclusive additional services for research, your expenses will be refunded. But only if you are in agreement, these schedules outside the normal hours of care for the making of the prosthesis will be performed.

You will have follow-up and assistance from the researchers throughout the study, including in case of need to stop the research, as well as indemnity guarantee against any recurring damages of the research.

All data related to you will be confidential and your identity will be kept confidential. The disclosure of the results will be carried out, preserving their identity, as names and / or documents of the participants in scientific meetings or articles to be published will not be published.

The necessary photographs will be only of the oral cavity and the total superior prosthesis, guaranteeing the preservation of its identity. The data obtained will be unique to this survey, and if they can be used in another survey, you will be notified again.

Your participation is not mandatory, and you may withdraw at any time by withdrawing your consent. Failure to participate in this research project will not prejudice any relationship with the researchers or the Dental School of Ribeirão Preto - USP.

You will receive a copy of this term, with the e-mail and telephone number of the researchers and the secretariat of the Research Ethics Committee (CEP) of FORP / USP. If you have any questions you can contact.

You can contact the Secretary of the Research Ethics Committee of the School of Dentistry of Ribeirão Preto - USP (Designated by CONEP for ethical follow-up of this research), in case you need any clarification or complaint. Phone: (16) 3315-0493, e-mail: cep@forp.usp.br and opening hours: from 8h to 12h, Monday to Friday (except holidays and optional points). You must identify yourself, but your identity will be kept in step.

Ribeirão Preto, Data _________________, 20____.

____

Prof.ª Dr.ª Cláudia Helena Lovato da Silva
Phone for contact: **(16) 3315-4006**
E-mail: **chl@forp.usp.br**

____

Camila Borba de Araújo
Phone for contact: **(16) 3315-4800**
E-mail: **camila.araujo@usp.br**

____

Adriana Barbosa Ribeiro
Phone for contact: **(16) 3315-4800**
E-mail: **adrianabarbosa84@bol.com.br**

____

Frank Lucarini Bueno
Phone for contact: **(16) 3315-4800**
Email: **franklucarini@usp.br**

I declare that I have understood the objectives, risks and benefits of my participation in the research and agree to participate.

____

Research Participant

General identity record:_____________________________

Phone for contact: (__)____________________

Address:____
